# Use of physiological knowledge to control the invasive sea lamprey (*Petromyzon marinus*) in the Laurentian Great Lakes

**DOI:** 10.1093/conphys/cox031

**Published:** 2017-05-30

**Authors:** Michael J. Siefkes

**Affiliations:** 1 Great Lakes Fishery Commission, 2100 Commonwealth Blvd., Suite 100, Ann Arbor, MI 48105, USA

**Keywords:** Conservation physiology, integrated pest management, invasive species, population control, sea lamprey

## Abstract

Sea lamprey (*Petromyzon marinus*) control in the Laurentian Great Lakes of North America is an example of using physiological knowledge to successfully control an invasive species and rehabilitate an ecosystem and valuable fishery. The parasitic sea lamprey contributed to the devastating collapse of native fish communities after invading the Great Lakes during the 1800s and early 1900s. Economic tragedy ensued with the loss of the fishery and severe impacts to property values and tourism resulting from sea lamprey-induced ecological changes. To control the sea lamprey and rehabilitate the once vibrant Great Lakes ecosystem and economy, the Great Lakes Fishery Commission (Commission) was formed by treaty between Canada and the United States in 1955. The Commission has developed a sea lamprey control programme based on their physiological vulnerabilities, which includes (i) the application of selective pesticides (lampricides), which successfully kill sedentary sea lamprey larvae in their natal streams; (ii) barriers to spawning migrations and associated traps to prevent infestations of upstream habitats and remove adult sea lamprey before they reproduce; and (iii) the release of sterilized males to reduce the reproductive potential of spawning populations in select streams. Since 1958, the application of the sea lamprey control programme has suppressed sea lamprey populations by ~90% from peak abundance. Great Lakes fish populations have rebounded and the economy is now thriving. In hopes of further enhancing the efficacy and selectivity of the sea lamprey control programme, the Commission is exploring the use of (i) sea lamprey chemosensory cues (pheromones and alarm cues) to manipulate behaviours and physiologies, and (ii) genetics to identify and manipulate genes associated with key physiological functions, for control purposes. Overall, the Commission capitalizes on the unique physiology of the sea lamprey and strives to develop a diverse integrated programme to successfully control a once devastating invasive species.

## Teaser

The sea lamprey is invasive to the Laurentian Great Lakes and managers have exploited the unique physiology of this basal vertebrate to control their populations and rehabilitate the Great Lakes ecosystem and fishery. Sea lamprey control is an example of conservation physiology successfully addressing a critical natural resource problem.

## Introduction

Conservation physiology is an emerging discipline that links physiological mechanisms in organisms to their changing environments in the context of conservation. Importantly, conservation physiology seeks to find solutions to complex conservation problems, one of which being the control of invasive species (Cooke et al., 2013). Perhaps one of the most successful examples of how physiological knowledge can be used in invasive species control is the sea lamprey (*Petromyzon marinus*) control programme in the Laurentian Great Lakes of North America (as noted in Madliger et al., 2016). Additionally, the sea lamprey control programme represents a good example of another key aspect of conservation physiology: using physiological knowledge to evaluate and improve management and conservation interventions (Cooke et al., 2013). Since the 1950s, many facets of the sea lamprey's unique physiology have been and are being used to devise and revise tactics to assess and control sea lamprey populations in the Great Lakes. This article highlights the sea lamprey control programme as a successful example of conservation physiology by describing the sea lamprey invasion of the Laurentian Great Lakes, the tactics used to control the sea lamprey, examples of emerging control tactics, and how physiological knowledge has been and could be used in the future to refine existing tactics and develop new tactics. For a more comprehensive review of the sea lamprey control programme and the research used to guide its development and application see Siefkes et al. (2013) and the proceedings of two sea lamprey international symposia (Canadian Journal of Fisheries and Aquatic Sciences 1980 37[11]; Journal of Great Lakes Research 2003 29[Supplement 1]).

## The sea lamprey invasion of the Laurentian Great Lakes

**Figure 1:**
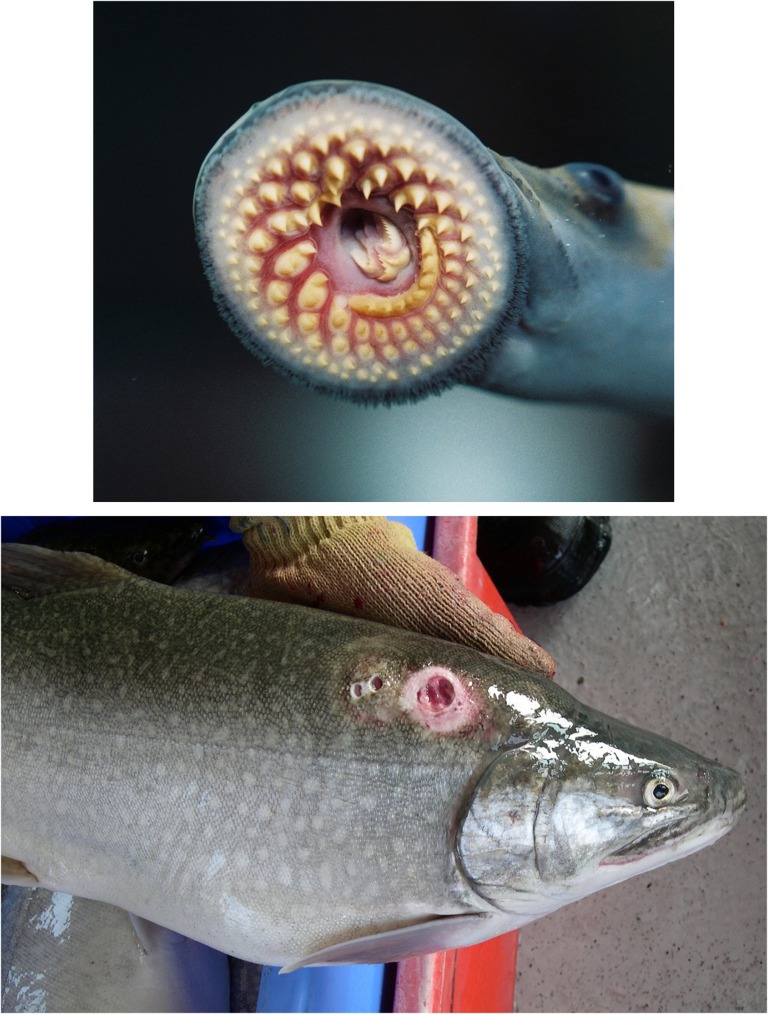
A sea lamprey highlighting the oral sucking disk and rasping tongue with pointy teeth (top). A sea lamprey wound on a lake trout (bottom). Photo credits: Top—T. Lawrence, Great Lakes Fishery Commission; bottom—P. Sullivan, Department of Fisheries and Oceans Canada.

The sea lamprey is a parasitic jawless fish native to the Atlantic Ocean (Fig. 1; Scott and Crossman, 1973; Hansen et al., 2016). Sea lamprey are anadromous, but also have the physiological ability to spend their entire life in fresh water making them capable of establishing landlocked populations. Landlocked sea lamprey, however, appear to have a reduced ability to acclimate to sea water (Beamish, 1980; Beamish, 1978). Sea lamprey use suction cup mouths with pointy teeth and rasping tongues to attach to and bore holes in the side of host fishes to feed on their blood and body fluids (Fig. 1). In the Atlantic Ocean, where sea lamprey coevolved with host fishes, sea lamprey are parasites and typically have little impact on host fish populations. In the Great Lakes, sea lamprey are much larger and prolific than smaller native lamprey and host fishes have not evolved to tolerate a parasite of this size. Consequently, in the Great Lakes, sea lamprey function more as a predator than a parasite, where a high proportion of host fishes die from sea lamprey attacks (Swink, 1990, 2003; Madenjian, 2008) and bioenergetics modelling has shown that a single sea lamprey can kill up to nearly 21 kg of fish (Kitchell and Breck, 1980; Swink, 2003).

Sea lamprey likely first gained access to the Great Lakes during the 1800s through the Erie Canal connecting Lake Ontario to the Hudson River, which drains to the Atlantic Ocean (Aron and Smith, 1971; Smith, 1995; Eshenroder, 2014). Sea lamprey invaded the other Great Lakes by the early to mid-1900s after the establishment and improvement of the Welland Canal, which provides a shipping route between Lakes Erie and Ontario bypassing the previously impassable Niagara Falls (Dymond, 1922; Applegate, 1950; Lawrie, 1970; Smith, 1971; Pearce et al., 1980; Smith and Tibbles, 1980).

**Figure 2:**
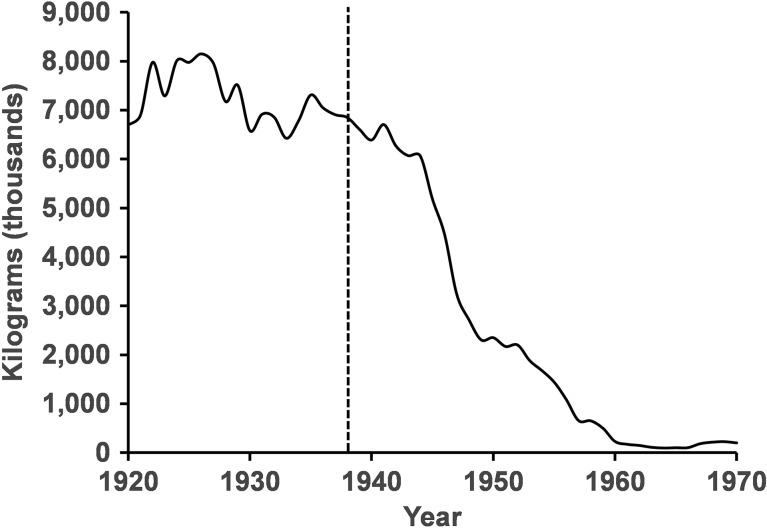
Lake trout production (harvest) for the Laurentian Great Lakes representing the crash of lake trout populations. The vertical dashed line is the date when sea lamprey were first observed in Lake Superior, the last of the lakes to be infested with sea lamprey. Lake trout data are from Baldwin et al. (2009).

After invading the Great Lakes, sea lamprey, with the help of overfishing and habitat degradation, caused the significant decline of many native fish species (Hile et al., 1951; Smith and Tibbles, 1980; Coble et al., 1990; Eshenroder and Burnham-Curtis, 1999; Hansen, 1999). Lake trout (*Salvelinus namaycush*), one of the most commercially valuable species and one of the top predators was especially hard-hit because it is the preferred host of the sea lamprey (Hansen et al., 2016). Sea lamprey select for larger hosts (Swink, 2003) such as lake trout and lake trout likely occupy the same temperatures as parasitic stage sea lamprey (Bergstedt, 2008). Consequently, lake trout were extirpated from Lakes Erie, Michigan and Ontario, nearly extirpated from Lake Huron, and driven to low abundance in Lake Superior (Berst and Spangler, 1973; Lawrie, 1978; Coble et al., 1990; Hansen, 1999; Eshenroder and Amatangelo, 2005; Muir et al., 2013). With the decline of lake trout (Fig. 2) came a predator/prey imbalance, which led to prey fish population spikes, particularly the non-indigenous alewife (*Alosa psuedoharengus*; Smith, 1970; Brown, 1972; O'Gorman and Stewart, 1999; Madenjian et al., 2011; O'Gorman et al., 2013), and subsequent die-offs that fouled marinas and beaches, ruined property values, and decimated local economies built on fishing and tourism (Scott and Crossman, 1973; Tanner and Tody, 2002).

**Figure 3:**
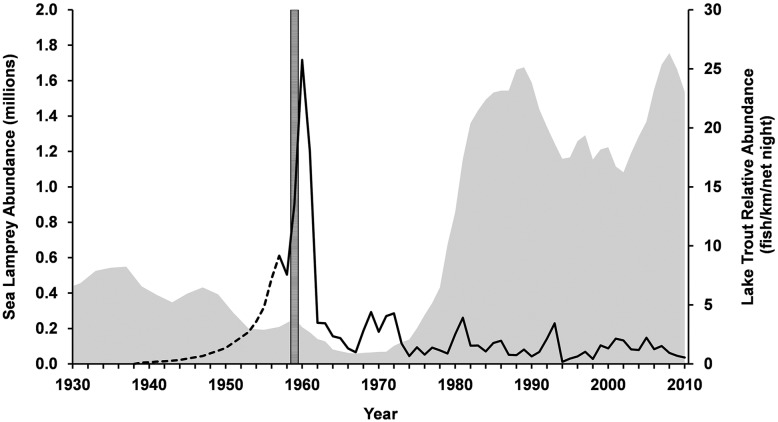
Lake Superior adult sea lamprey abundance estimates (solid black line) compared with lake trout relative abundance (fish/km/net night) in Michigan waters of Lake Superior (gray shading). The dashed black line depicts a hypothetical sea lamprey population increase from the year sea lamprey were first discovered in Lake Superior (1938) to the year when sea lamprey abundance was first modelled using trap catch data (1957). The segmented vertical bar represents the year lampricide treatments began in Lake Superior (1959). Lake trout data are from S. Sitar, Michigan Department of Natural Resources and M. Wilberg, The University of Maryland Center for Environmental Science and sea lamprey data are from the Great Lakes Fishery Commission.

To attempt to reverse the devastating impacts of the sea lamprey invasion, the federal governments of Canada and the United States established the Great Lakes Fishery Commission (Commission) by treaty in 1955 to coordinate fisheries management, implement a research programme to promote the rehabilitation of Great Lakes fisheries, and develop and implement a sea lamprey control programme. By the early 1960s, a sea lamprey control programme was developed through Commission-sponsored research. Annual application of sea lamprey control that continues today has reduced sea lamprey abundance from peak levels by nearly 90% across the lakes and allowed for the rehabilitation of fish stocks, in particular lake trout (Fig. 3). Successful sea lamprey control coupled with coordinated fisheries management, both supported by research, has rehabilitated a Great Lakes ecosystem capable of supporting an economy based on fishing and tourism that is currently valued at more than $7 billion annually (Southwick Associates, 2012). The sea lamprey control programme represents a remarkable success in invasive species control for one of the world's largest freshwater ecosystems and is perhaps the most successful programme of its kind.

### Successful sea lamprey control requires physiological knowledge

**Figure 4:**
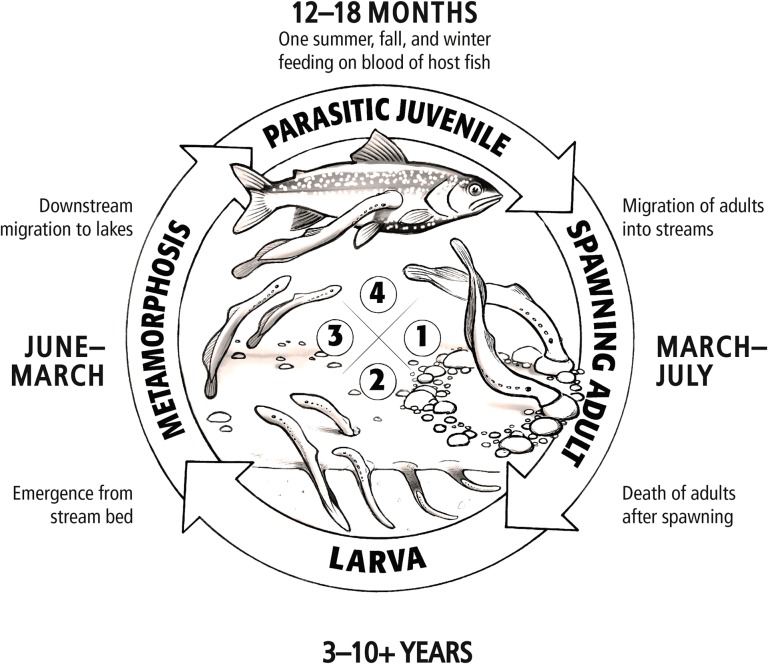
The sea lamprey life cycle. Image credit: Great Lakes Fishery Commission.

**Figure 5:**
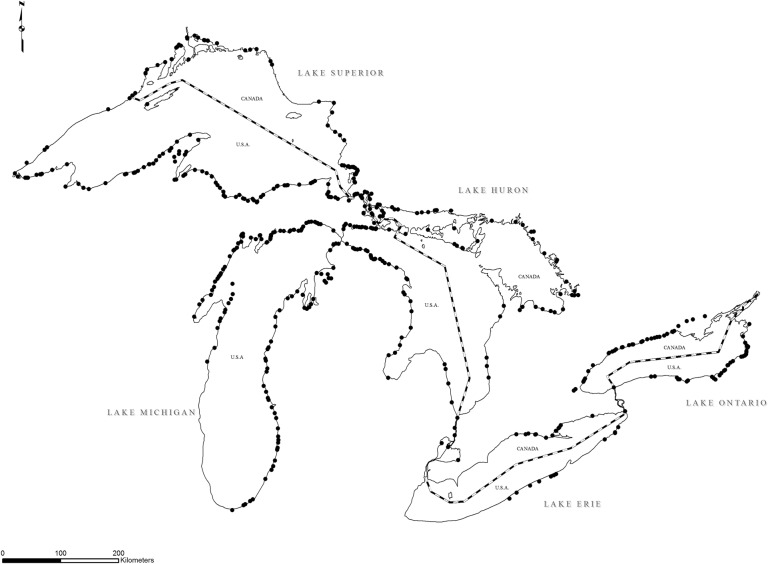
Approximately 500 Great Lakes streams have been infested with sea lamprey. Image credit: Great Lakes Fishery Commission.

Success of any pest control programme lies in gaining an intimate understanding of the biology and ecology of the target organism. After the sea lamprey invasion, scientists began collecting this knowledge by first determining the sea lamprey life cycle (Fig. 4) and distribution in the Great Lakes (Fig. 5). Sea lamprey begin life in late spring/early summer as filter-feeding larvae (also called ammocoetes) that reside in their natal streams for 3 to possibly more than 10 years (Potter, 1980; Purvis, 1980) and larval sea lamprey were found to reside in ~500 Great Lakes streams (Applegate, 1950). Starting at a length of ~120 mm (Potter, 1980; Purvis, 1980), larval sea lamprey begin a dramatic metamorphosis during the summer where they develop eyes, a suction cup mouth and rasping tongue with pointy teeth, and migrate to the lakes to feed on fishes (Youson, 2003; 1980). After feeding for 12–18 months (Hardisty and Potter, 1971; Farmer, 1980), sea lamprey detach from their host, migrate to and up a suitable spawning stream, reproduce and die (Hardisty and Potter, 1971; Larsen, 1980).

Scientists at the time realized the best chance to control sea lamprey was during its stream-dwelling stages as either larvae or adults where they are relatively concentrated and their distributions are known. With this knowledge in hand, scientists began conducting research to understand the physiological vulnerabilities of the sea lamprey in hopes to ultimately develop sea lamprey control techniques to exploit these vulnerabilities. Since the 1950s, a suite of successful sea lamprey control techniques have been developed and refined with ongoing research. The control techniques in use today include lampricides, barriers to spawning migrations, traps and sterile male releases. These techniques are highlighted next along with the key physiological research responsible for their development and refinement.

### Understanding toxicology to develop lampricides

**Figure 6:**
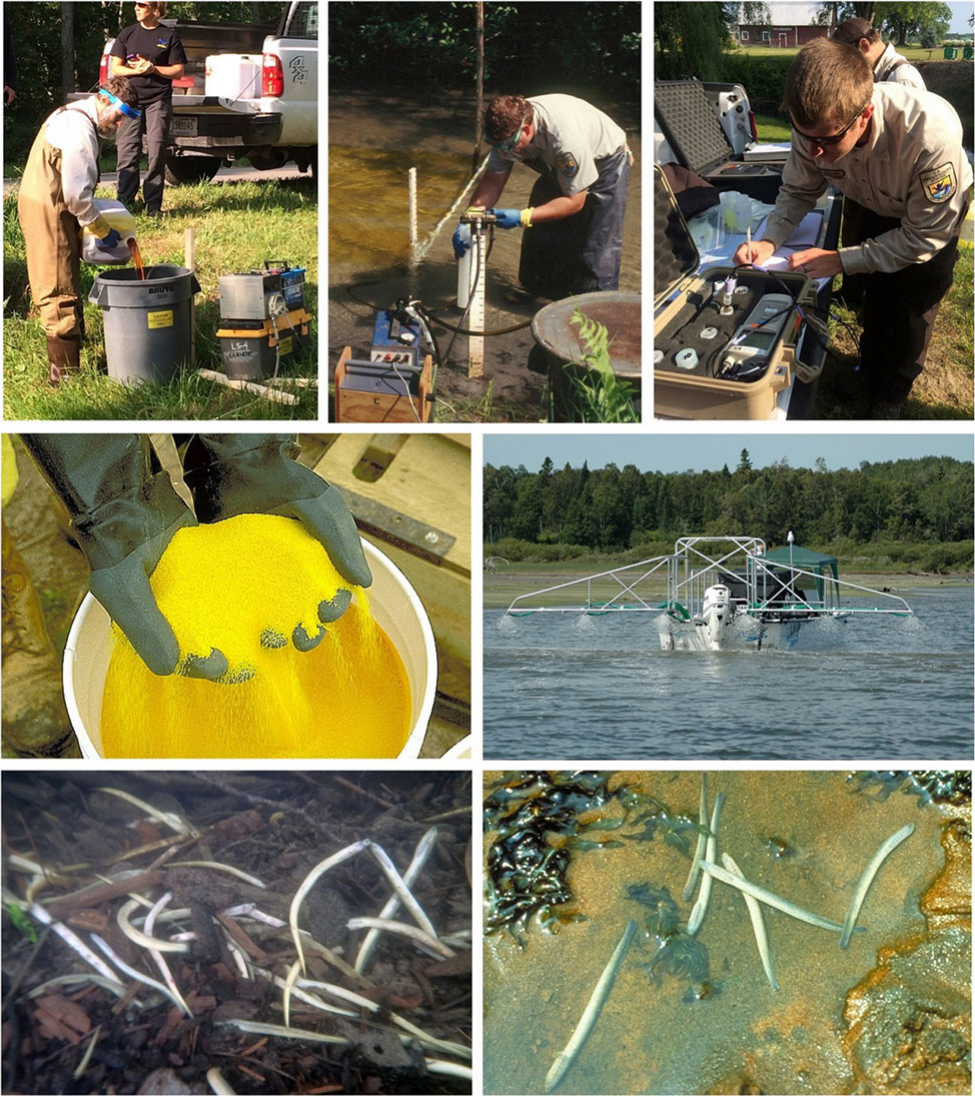
3-Trifluoromethyl-4-nitrophenol (TFM) is the primary lampricide and is used to target larval sea lamprey residing in Great Lakes streams (top three pictures). 2′,5-Dichloro-4′-nitrosalicylanilide (niclosamide) is used as an additive to TFM during larger treatments, which reduces costs, but maintains the effectiveness and selectivity of the treatments. A time-release granular formulation of niclosamide is used to treat infested connecting waterways between the lakes and estuaries of infested streams (middle two pictures). Sea lamprey larvae are killed (bottom two pictures) before they can harm fishes. Photo credits: Top left and right—M. Siefkes, Great Lakes Fishery Commission; top centre and middle—T. Lawrence, Great Lakes Fishery Commission; bottom—Great Lakes Fishery Commission.

During the 1950s, scientists began searching for pesticides that could kill larval sea lamprey in their natal streams before they metamorphosed and migrated to the lakes to feed on fishes. Successful development of any pesticide lies in its selective toxicity to the target organism. For sea lamprey, the pesticide (lampricide) should kill larval sea lamprey, but have minimal effects on other species that reside in the same habitats. During the development of lampricides, nearly 10 000 mostly organic compounds were screened for their selective toxicity to sea lamprey (Howell et al., 1980). Two compounds, 3-trifluoromethyl-4-nitrophenol (TFM) and 2′,5-dichloro-4′-nitrosalicylanilide (niclosamide) were identified as promising lampricide candidates. TFM was found to be selectively toxic and became the primary lampricide used to treat sea lamprey-infested streams across the Great Lakes basin (Fig. 6). Niclosamide was found to have similar toxicity to sea lamprey and non-target fishes (Dawson, 2003), but was less expensive than TFM (Howell et al., 1980). Niclosamide, however, was found to be effective when added in small amounts to TFM during larger treatments by reducing TFM use and thus costs, but maintaining the effectiveness and selectivity of the treatments (Marking and Hogan, 1967; Dawson et al., 1977; Howell et al., 1980; Dawson, 2003). A time-release granular formulation of niclosamide was also developed for treating infested connecting waterways between the lakes (very large rivers) and estuaries of infested streams (Howell et al., 1980; Dawson, 2003; Fig. 6). Treating these larger areas with niclosamide was more cost effective than treating with TFM and the time-release formulation of niclosamide restricts the toxicity to the lower portion of the water column (Dawson, 2003) allowing non-target fishes the opportunity to avoid the lampricide.

Early speculation, based on studies from similarly structured compounds, was that TFM disrupts oxidative phosphorylation leading to rapid energy depletion (Applegate et al., 1966; Niblett and Ballantyne, 1976; Howell et al., 1980), but it was not until recently that this hypothesis was more definitively tested (Wilkie et al., 2007; Birceanu et al., 2009, 2011). Theoretically, if TFM disrupts oxidative phosphorylation, energy production would shift from the aerobic to anaerobic pathways, resulting in a decrease in glycogen and an increase in anaerobic waste products in tissues. Experiments showed that TFM exposure decreased glycogen concentrations in the brain, liver and muscle, while increasing lactate in the same tissues (Birceanu et al., 2009). These results support the hypothesis that TFM disrupts oxidative phosphorylation leading to a mismatch in energy supply and demand and a build-up of toxic anaerobic waste products ultimately leading to death. The mode of action of niclosamide has not yet been fully described, but may be similar to that of TFM (Dawson, 2003).

Selectivity of TFM appears to be associated with differences in the ability of sea lamprey and other fishes to detoxify and excrete TFM after biotransformation through the process of glucuronidation (Howell et al., 1980; Hubert, 2003). The most abundant metabolite of TFM in fishes was shown to be the glucuronide conjugate (Hubert, 2003; Lech and Costrini, 1972; Lech, 1973; Lech and Statham, 1975; Kane et al., 1994; Hubert et al., 2005). Therefore, glucuronide conjugation of TFM and excretion via bile was concluded to be the mechanism of detoxification in fishes (Lech et al., 1973; Lech, 1973; Hunn and Allen, 1975a, b; Schultz et al., 1979). In contrast, glucuronide conjugates of TFM are not abundant in sea lamprey (Lech and Statham, 1975; Kane et al., 1994), indicating a limited ability to conjugate TFM with glucuronic acid, making TFM harder to excrete, causing TFM to build in concentration in the body, and ultimately causing death through disruption of oxidative phosphorylation. This inability to detoxify TFM was found to be caused by a lower glucuronyl transferase activity in sea lamprey compared to other fishes like rainbow trout (*Oncorhynchus mykiss*; Lech and Statham, 1975; Kane et al., 1994). Niclosamide metabolism in fishes is less understood, but niclosamide is known to be less selective than TFM (Dawson, 2003) and its detoxification produces both the sulphate ester and the glucuronide conjugate in rainbow trout (Statham and Lech, 1975; Dawson, 2003; Hubert et al., 2005) suggesting the metabolic pathway for niclosamide may be different than the pathway for TFM. Although TFM shows relative selectivity to lamprey, the selectivity ratio for TFM is not high; the concentration of TFM that kills non-target fishes is between 2 and 10 times that used to kill sea lamprey—some insecticides have a selectivity ratio as high as 1000 (Howell et al., 1980). Therefore, and because of the lower selectivity of niclosamide, extreme care must be taken when applying lampricides to ensure lethal concentrations are achieved for sea lamprey and not non-target fishes.

Additional physiological research identified key environmental variables that affect TFM toxicity and selectivity. In particular, pH was found to have an inverse relationship with TFM toxicity (LeMaire, 1961; Hunn and Allen, 1974; Howell et al., 1980; Bills et al., 2003). TFM toxicity was also found to be lower in more alkaline water and that the pH/TFM toxicity relationship was exacerbated by higher alkalinity (Dawson et al., 1975; Bills et al., 2003). The mechanism behind this phenomenon is likely TFM speciation, with the lipid-soluble, free phenol form of TFM, which is more easily absorbed across biological membranes, being more abundant at lower pH and alkalinity (Howell et al., 1980). The ionic, phenolate forms of TFM, which cannot cross biological membranes, becomes more abundant at higher pH and alkalinity (Howell et al., 1980). The solubility of niclosamide decreases with lower pH, but a decrease in toxicity at lower pH values was not observed (Dawson et al., 1977). Since Great Lakes streams have diverse water chemistries and their pH can have dramatic diel shifts due to aquatic plant respiration during non-daylight hours (Bills et al., 2003), the influence of pH and alkalinity on TFM toxicity is accounted for when planning and conducting lampricide treatments.

The original discovery of TFM and niclosamide led to the creation of a highly effective, large-scale, lampricide application protocol, which has been refined over 60+ years based on further physiological research to increase its effectiveness and selectivity. Even though much has been learned about the mode of action of lampricides, particularly TFM, elucidation of exactly how TFM and niclosamide disrupt oxidative phosphorylation could provide further information to enhance current lampricide treatments and insights into the identification and development of more effective and selective lampricides. Although the effects of lampricides on stream macro-invertebrates has been studied (Gilderhus et al., 1975; Maki et al., 1975; Gilderhus and Johnson, 1980; Waller et al., 2003; Weisser et al., 2003; Boogaard and Rivera, 2011; Boogaard et al., 2015; Newton et al., 2017), research addressing the non-target effects of lampricides has mostly focused on fishes (Dahl and McDonald, 1980; Boogaard et al., 2003). A more complete understanding of lampricide effects on a suite of aquatic organisms would provide insights on how to be more effective at targeting sea lamprey while minimizing impacts to non-target organisms through the revision of protocols or the development of new lampricides. Additionally, although no evidence exists for it, lampricide resistance is a concern for the sea lamprey control programme (Dunlop et al., 2017). Further physiological research to support the development of new lampricides that target different mechanisms will help mitigate the risk of developing lampricide resistance. Overall, lampricides are the backbone of the sea lamprey control programme and are largely responsible for the ~90% decline in sea lamprey abundance from peak levels (Pearce et al., 1980; Smith and Tibbles, 1980; Heinrich et al., 2003; Larson et al., 2003; Morse et al., 2003; Lavis et al., 2003a), which has ultimately led to the rehabilitation of Great Lakes fish communities, the ecosystem and the related economy.

### Understanding swim performance and motivation to design barriers to spawning migration and traps

**Figure 7:**
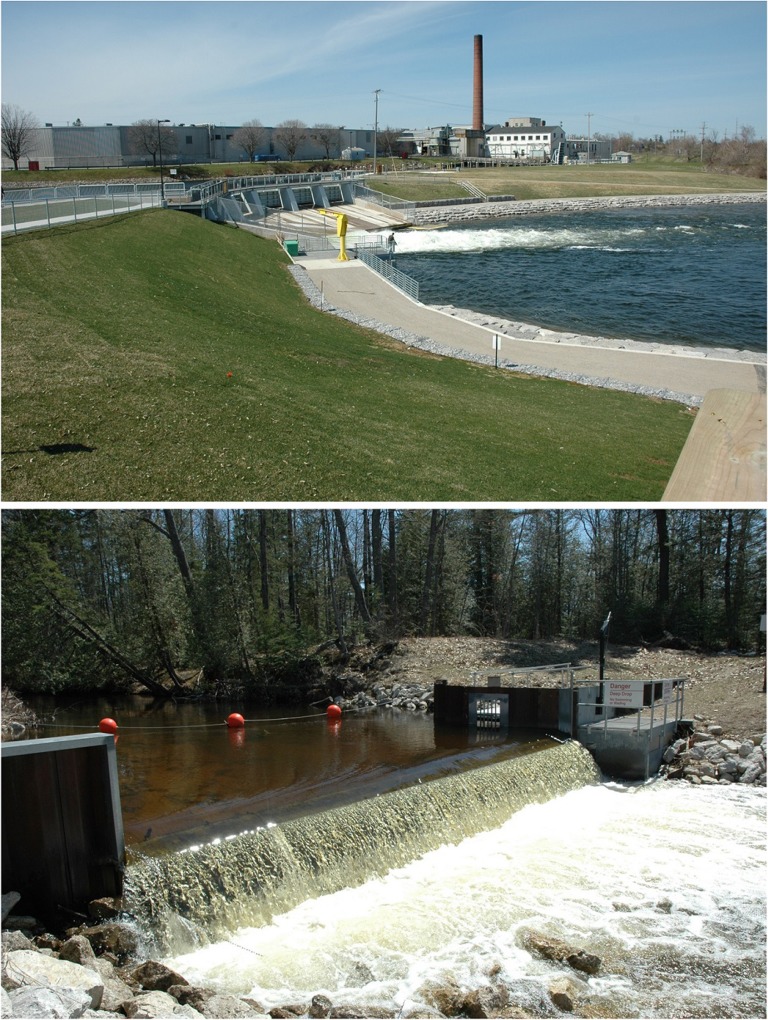
Sea lamprey barriers reduce the length of streams that need lampricide treatment. Hundreds of sea lamprey barriers are dams built for other purposes (hydropower, flood control, etc.) that fortuitously block adult sea lamprey spawning migrations (top). Additionally, barriers have been purposely built or modified to block adult sea lamprey migrations (bottom). Photo credits: M. Siefkes, Great Lakes Fishery Commission.

Before the development of lampricides, some of the earliest control tactics exploited the adult sea lamprey's strong instinct to swim upstream (Manion and Hanson, 1980), including barriers to spawning migrations and removal of adult sea lampreys with traps (Hunn and Youngs, 1980). Sea lamprey barriers are an integral part of the sea lamprey control programme because they reduce the length of streams that need lampricide treatment. Hundreds of sea lamprey barriers are dams built for other purposes (hydropower, flood control, etc.) that fortuitously block adult sea lamprey spawning migrations (Smith and Tibbles, 1980; Siefkes et al., 2013; Fig. 7). The Commission also maintains a network of 73 barriers that were purposely built or modified to block adult sea lamprey migrations (Lavis et al., 2003b; Siefkes et al., 2013; Fig. 7). Like lampricides, selectivity is desirable for sea lamprey barriers, which should block sea lamprey and not non-target fishes. Many dams built for other purposes that serve as sea lamprey barriers are not selective. In an attempt to achieve some selectivity, purpose-built barrier designs have been informed by swimming performance research on sea lamprey and non-target fishes. In general, sea lampreys are relatively poor jumpers and swimmers (Beamish, 1978; Youngs, 1979; Reinhardt et al., 2009; Almeida and Quintella, 2013). Therefore, sea lamprey barriers do not have to be tall structures and most are low-head structures designed to maintain only a 45 cm drop during the spawning season (Hunn and Youngs, 1980). This design allows for the passage of jumping fishes, although non-jumping fishes, many of which are critically important to the Great Lakes ecosystem and fishery, are still blocked (Hunn and Youngs, 1980; Dodd et al., 2003).

**Figure 8:**
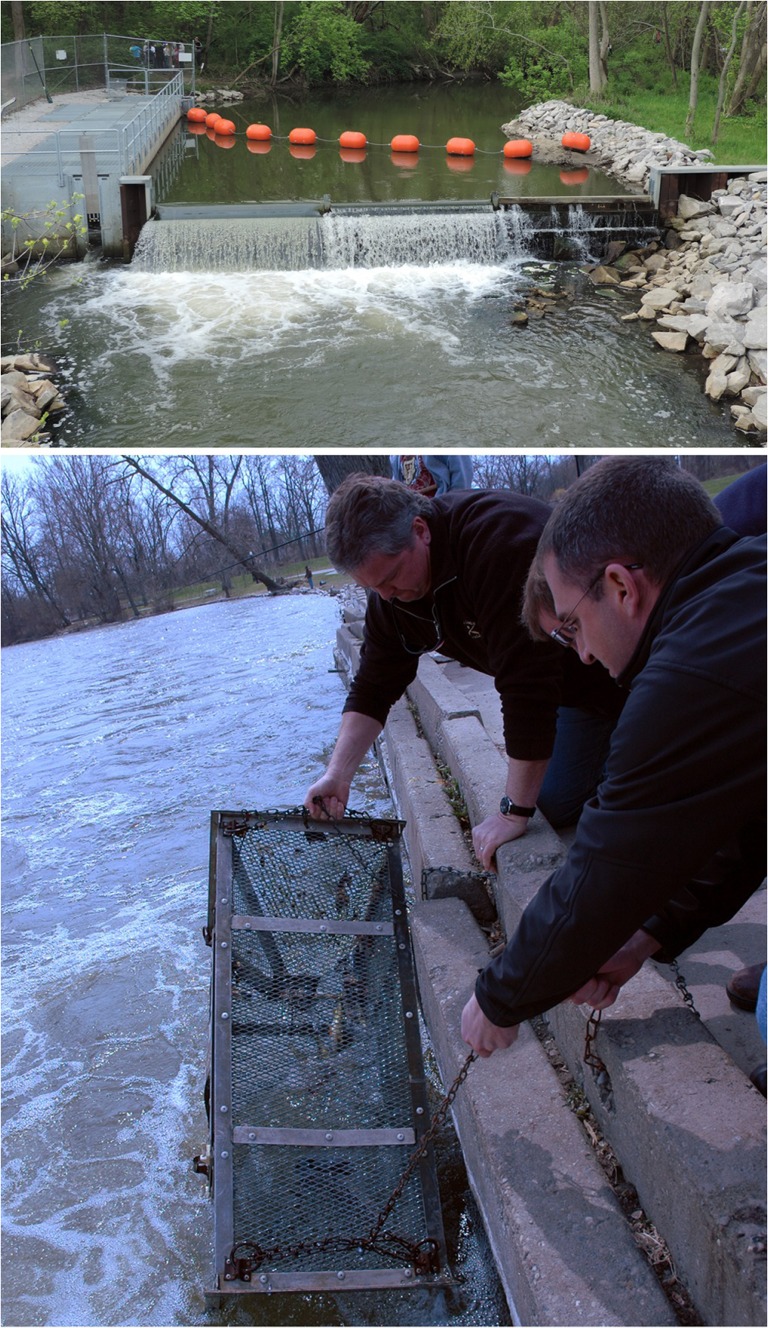
Most purpose-built sea lamprey barriers have adult sea lamprey traps either integrated into their design (top; left side of barrier) or placed along their face (bottom). Although reproductive potential in a stream can be reduced by removing captured adults, high fecundity and compensatory mechanisms prevent significant impact to the recruitment of parasitic juveniles to the lakes. Trapping does serve as a critical assessment tool and holds high promise as a future control technique if tactics that increase trapping efficiencies are developed. Photo credits: Top—M. Ryan, Great Lakes Fishery Commission; bottom—A. Muir, Great Lakes Fishery Commission.

Most purpose-built sea lamprey barriers have adult sea lamprey traps either integrated into their design or placed along their face (Fig. 8). These traps are designed like large minnow traps (Schuldt and Heinrich, 1982) and take advantage of the sea lamprey's persistence in moving upstream during spawning migrations and the barrier's ability to congregate sea lamprey, increasing the probability of trap encounter and entrance. When adult sea lamprey encounter a barrier, they probe the face of the barrier looking for a passage upstream. Consequently, some adult sea lamprey find and enter the trap during their search. Although reproductive potential in a stream can be reduced by removing captured adults from the spawning population, removing enough adults to overcome high fecundity (Manion and Hanson, 1980; a single female sea lamprey can produce up to 100 000 eggs), compensatory mechanisms such as increased larval survival at lower spawning densities, and density-independent variation in survival (Jones et al., 2003) enough to impact the recruitment of parasitic juveniles to the lakes has been unsuccessful to date. Nevertheless, trapping serves as a critical assessment tool to gauge sea lamprey control programme success by tracking adult sea lamprey abundance trends in each lake over time (Mullett et al., 2003) and holds high promise as a future control technique once scientific breakthroughs to increase trapping efficiency are achieved (e.g. see Understanding chemosensory cues to manipulate behaviours below).

Sea lamprey barriers are critically important to the sea lamprey control programme. In fact, sea lamprey control would likely not exist if it were not for sea lamprey barriers, especially those built for other purposes that protect thousands of kilometres of streams from sea lamprey infestation. The increase in the amount of lampricide needed to control sea lamprey in the absence of these barriers would likely not be economically feasible. Nevertheless, the need for sea lamprey barriers can conflict with the ecosystem and fishery restoration goals of allowing native fish passage to historical habitats to rehabilitate their populations. Additionally, although traps serve a critical assessment function for sea lamprey control, their potential as a control tactic has not yet been fully realized (Siefkes et al., 2013). Research is ongoing to better understand adult sea lamprey behaviour and swimming performance as they relate to barriers and traps (Quintella et al., 2004, 2009; McLaughlin et al., 2007; Bravener and McLaughlin, 2013; Holbrook et al., 2015) and further research is needed to better understand the stress imposed by barriers and fish passage structures on both sea lampreys and non-target fishes and how this stress may impact fitness. Results from this research and future research could provide insight on how to balance the potentially conflicting goals of aquatic habitat connectivity and invasive species control, and develop more effective selective fish passage and trapping techniques.

### Understanding reproductive ecology, physiology and mechanisms of DNA damage to implement sterile male releases

The release of sterilized males, a technique first developed for insects (Knipling, 1968), has been used as a sea lamprey control tactic (Schleen et al., 2003; Twohey et al., 2003a). The concept behind the technique is that the reproductive potential of a population is reduced by saturating the population with sterilized males that compete successfully with fertile males for mates. The upside of the technique for sea lamprey is that it is species-specific and environmentally benign compared to lampricides and barriers. The downside is that the technique will only work if enough sterilized males can be released to overwhelm the fertile male population (Twohey et al., 2003a). Due to the limited number of males available for sterilization, the technique will likely only be effective on low density populations and in streams where adult trapping is highly efficient.

Development of the sterile male release technique began in the 1970s with the discovery that P,P-bis (1-aziridinyl)-*N*-methylphosphinothioic amide (Bisazir; Chang et al., 1970) was an effective sea lamprey sterilant (Hanson and Manion, 1978; Hanson, 1981). Bisazir causes sterility in sea lamprey by damaging the genetic material in their sperm (Hanson, 1990), however, the ability of sterilized males to fertilize eggs is not affected. Nevertheless, nearly all eggs fertilized by sterilized males die before hatching (Ciereszko et al., 2002). Importantly, Bisazir was also shown not to affect male competitiveness or suppress critical spawning behaviours (Hanson and Manion, 1980; 1978), and was later found not to affect sex pheromone production in males (Siefkes et al., 2003). Maintaining the competitiveness and attractiveness of sterilized males is critical to the success of the technique.

The sterile male release technique was first field tested in Lake Superior streams and the St. Marys River from 1991 to 1996 (Kaye et al., 2003; Twohey et al., 2003a) and was later deployed entirely in the St. Marys River from 1997 to 2011 (Bravener and Twohey, 2016). The St. Marys River, the large connecting waterway between Lakes Superior and Huron, was selected for deployment of the technique because of its large, uncontrolled larval sea lamprey population (Schleen et al., 2003) and the size of the waterway prevented treatment with the main lampricide TFM due to the large amount and subsequent cost of TFM needed for the treatment. Although the technique likely had an impact on the larval population in the St. Marys River (Bergstedt et al., 2003; Schleen et al., 2003; Twohey et al., 2003a; Bravener and Twohey, 2016), increasing larval sea lamprey populations caused a re-evaluation of the technique, which showed the application of the granular form of niclosamide alone would be the most effective control technique for the St. Marys River (Jones et al., 2015). Subsequently, the technique was discontinued in the St. Marys River in 2011.

**Figure 9:**
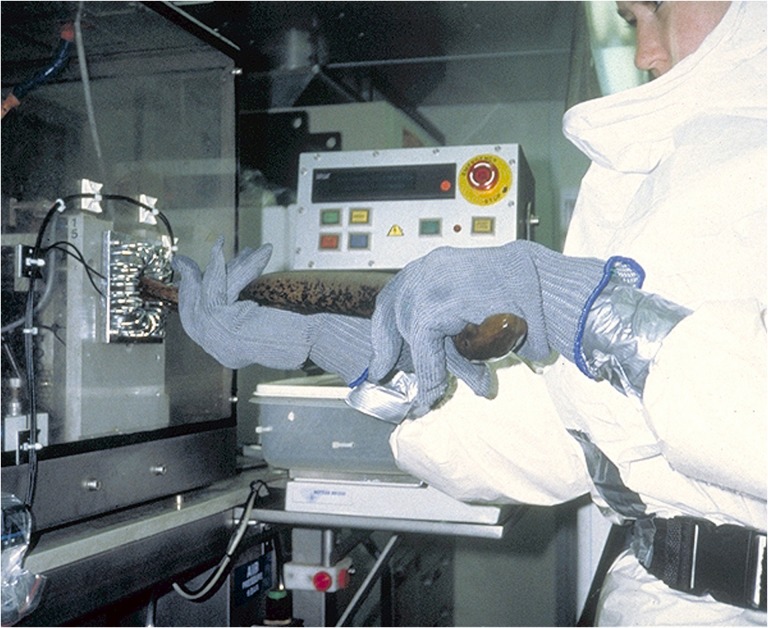
The release of sterilized males has been developed as a potential environmentally benign, species-specific sea lamprey control tactic. The technique requires a dedicated facility and auto-injector (pictured) due to the mutagenic properties of the sterilant P,P-bis (1-aziridinyl)-*N*-methylphosphinothioic amide (Bisazir). Photo credit: Great Lakes Fishery Commission.

Although the sterile male release technique is currently not being deployed, it still remains a viable sea lamprey control option for low density populations. Valuable lessons were learned during the technique's previous deployments (Kaye et al., 2003; Twohey et al., 2003a; Bravener and Twohey, 2016) and a re-evaluation of the technique on a smaller, lower density stream is currently being considered. Importantly, a strong understanding of the population dynamics for both the adult and larval life stages in the target stream is needed to evaluate the success of the technique. Also, a major limitation of the technique is the mutagenic nature of Bisazir. A dedicated facility and auto-injector were constructed to contain the threat of and minimize staff exposure to Bisazir (Twohey et al., 2003a; Fig. 9). Consequently, the technique would only be cost-feasible to deploy in streams in close proximity to the sterilization facility. Developing portable sterilization units that could replicate the safeties of the facility or identifying a more benign sterilant such as hormones (Sower, 2003), anti-fertility compounds (Ciereszko et al., 2003), other agents that cause sterility through DNA damage (Hanson, 1990; Ciereszko et al., 2005), and the development of RNA interference sterility techniques (Whyard et al., 2015) would allow for expansion of the technique beyond the current geographic limitations.

## Emerging control tactics

Current sea lamprey control relies exclusively on lampricides and barriers, two technologies that can have significant negative impacts on non-target organisms. Even though ongoing physiological research continues to sharpen the effectiveness and selectivity of these techniques, there is a strong desire to develop new, innovative techniques that could provide levels of effectiveness and selectivity yet to be seen and prevent catastrophic population increases if one of the current techniques is rendered ineffective (e.g. development of lampricide resistance). Two avenues of research, chemosensory cues and genetics, have shown strong potential for the development of effective and selective sea lamprey control tactics and are discussed below. Other aspects of sea lamprey, non-target organism and host fish physiologies are being explored with the intent of further integrating and advancing sea lamprey control, but are not highlighted in this article.

### Understanding chemosensory cues to manipulate behaviours

The use of chemosensory cues such as pheromones and alarm substances has long been suspected of potentially being useful for sea lamprey control purposes (Teeter, 1980; Li et al., 2003, 2007; Twohey et al., 2003b; Wagner et al., 2011; Buchinger et al., 2015). Although monorhinic, the olfactory organ of the juvenile and adult sea lamprey is relatively large with numerous longitudinal folds (Kleerekoper and van Erkel, 1960) and the proportion of the post-larval brain dedicated to olfaction is high among vertebrates (Stoddart, 1990). Additionally, olfaction appears to be critically important to the reproductive fitness of this semelparous animal as adult sea lampreys produce and respond to pheromones and alarm cues released by multiple life stages to coordinate spawning migration and reproduction (Teeter, 1980; Li et al., 2002; Sorensen et al., 2005; Wagner et al., 2011). Therefore, manipulating adult sea lamprey behaviours or physiological processes using chemosensory cues or disrupting chemosensory communication could potentially lead to reproductive failure and decreased sea lamprey populations.

**Figure 10:**
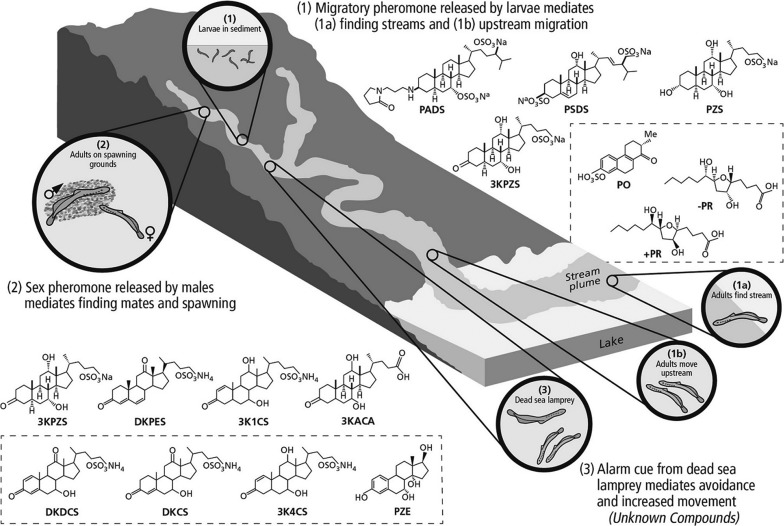
Current understanding of sea lamprey reproductive chemosensory communication. Adult sea lamprey use a migratory pheromone released by (1) larvae to (1a) find and (1b) migrate up suitable spawning streams. (2) Adults then use a sex pheromone released by males to coordinate spawning. (3) An alarm cue from dead sea lamprey mediates avoidance behaviours and increased movement in adults. Structures of compounds released by (1) larvae and (2) adult males that are hypothesized to function as pheromones are displayed; dashed boxes denote compounds that have not yet been shown to induce behavioural or physiological activity. Petromyzonamine dimonosulfate (PADS); petromyzosterol disulfate (PSDS); petromyzonol sulphate (PZS); petromyzonin (PO); 3-keto petromyzonol sulphate (3KPZS); −petromyroxol (−PR); +petromyroxol (+PR); 3,12-diketo 4,6-petromyzonene 24-sulphate (DKPES); 3-keto allocholic acid (3KACA); 7,12,24-trihydroxy 3-keto 4-choline 24-sulphate (3K4CS); 7,24-dihydroxy 3,12- diketo 1,4-choline 24-sulphate (DKDCS); 7,24-dihydroxy 3,12-diketo 4-choline 24-sulphate (DKCS); 7,12,24- trihydroxy 3-keto 1-choline 24-sulphate (3K1CS); petromyzesterol (PZE).

The adult sea lamprey's reproductive journey begins with finding suitable spawning streams while navigating vast expanses of open water. Adult sea lamprey do not home (Bergstedt and Seelye, 1995; Waldman et al., 2008), but instead use a migratory pheromone released by larval sea lamprey to help find spawning streams (Teeter, 1980; Sorensen et al., 2005; Fig. 10); the presence of larval odour indicates that a stream is suitable for reproduction. Several bile acids released by larval sea lamprey (Fig. 10), including petromyzonol sulphate (PZS), petromyzonamine disulfate (PADS), petromyzosterol disulfate (PSDS) and 3-keto petromyzonol sulphate (3KPZS) influence the behaviour of migrating adult sea lamprey in the lab (Bjerselius et al., 2000; Sorensen et al., 2005; Johnson et al., 2013) and appear to partially mediate stream-finding behaviours in lakes near the mouths of streams (Meckley et al., 2014). Nevertheless, these compounds do not appear to comprise the complete migratory pheromone released by larval sea lamprey as none have been shown to induce upstream movement of migrating adults once they enter a stream (Meckley et al., 2012). Thus, key components of the migratory pheromone remain unknown. Several putative migratory pheromone compounds have been recently identified, but have not yet been behaviourally evaluated (Li et al., 2014, 2013a; Fig. 10).

Sea lamprey complete sexual maturation while migrating within streams. Male sea lamprey typically precede females in migrating to spawning areas consisting of gravel/cobble substrate and cool, well-oxygenated water (Applegate, 1950). Males begin to construct nests by moving rocks to create a horseshoe impression in the substrate shortly after their arrival. Once females are sexually mature, they join the males on a nest to spawn. Coordination of these final steps in the reproductive process is guided by a sex pheromone released by sexually mature males (Li et al., 2002; Fig. 10). At least four bile acids, including 3KPZS (also released by larvae), 3-keto allocholic acid (3KACA), diketo petromyzonene sulphate (DKPES) and 3-keto 1-ene petromyzonol sulphate (3K1CS) are thought to comprise the male sex pheromone (Fig. 10). 3KPZS and 3KACA have been shown to help elicit sexual maturation in both males and females (Chung-Davidson et al., 2013a, b) thus synchronizing the reproductive state of conspecifics. 3kPZS also has a behavioural function, inducing upstream swimming in sexually mature females (Li et al., 2002; Siefkes et al., 2005; Johnson et al., 2009) to aid them in finding nesting males, and eliciting nest construction and pair maintenance behaviours (Johnson et al., 2012) to coordinate the final act of reproduction. Recently, DKPES and 3K1CS were shown to further assist females in finding males (Li et al., 2013b; Johnson et al., 2014) and DKPES enhanced the attractiveness of 3kPZS when the two compounds are mixed (Li et al., 2013b). Despite the ability of these compounds to induce behavioural and physiological responses in conspecifics, the complete male odour is significantly more attractive to mature females, indicating that key components of the sex pheromone remain unidentified.

Most recently, investigations of the importance of alarm cues to sea lamprey reproductive fitness have begun. Alarm cues can be odours produced by both dead or alive conspecifics and heterospecifics and are hypothesized to function in sea lamprey as a way to assess risk, for instance, predatory risk, the risk of choosing poor habitat conditions and the risk of choosing streams in which spawning has already occurred (sea lampreys die after spawning). In support of this hypothesis, conspecific odours from deceased sea lamprey have been shown to induce avoidance and flight responses in adult conspecifics (Bals and Wagner, 2012; Fig. 10). Additionally, 2-phenylethylamine HCl (PEA), a putative predator cue in rodents (Ferrero et al., 2011), has been shown to induce avoidance responses in adult sea lamprey (Di Rocco et al., 2014; Imre et al., 2014). Given that the exploration of sea lamprey alarm cues is a recent endeavour, the conspecific and heterospecific alarm cues responsible for the above behaviours (except PEA) remain unidentified.

**Figure 11:**
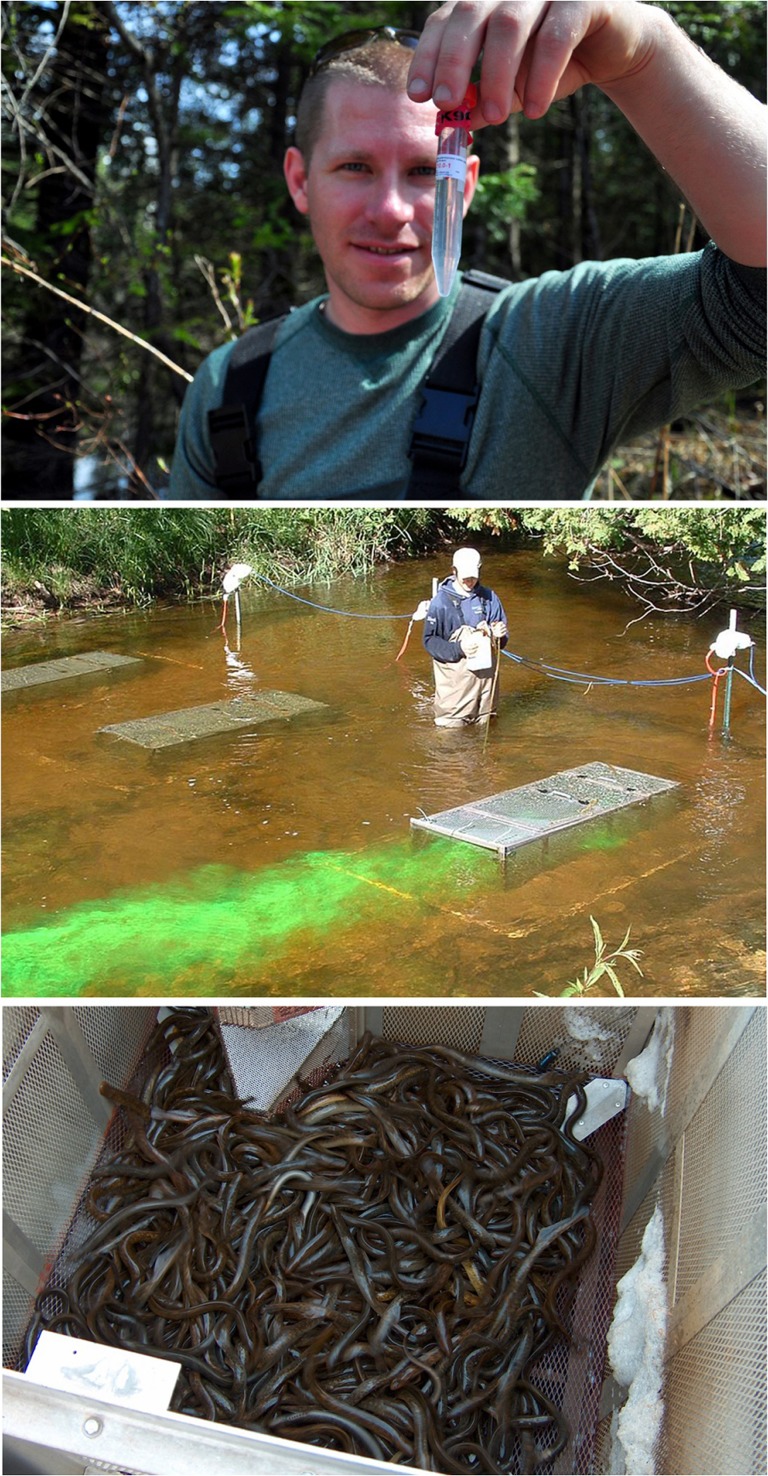
Pheromone-baited trapping is the only method using chemosensory cues that has been explored on a management scale and could potentially increase trapping efficiencies enough to reduce sea lamprey recruitment. Pictures from top to bottom: a vile of 3-keto petromyzonol sulphate (3KPZS), the male sex pheromone component used in management scale testing; dye test during a chemosensory cue trapping experiment; a sea lamprey trap baited with 3KPZS filled with sea lampreys. Photo credits: Top—A. Miehls, Great Lakes Fishery Commission; middle—M. Moriarty; Bottom, Great Lakes Fishery Commission.

Chemosensory cues have been proposed to be integrated into the sea lamprey control programme in several ways, including trapping of adults, redistribution of adults to areas that would reduce fitness (poor habitats, high lampricide and trapping effectiveness), communication disruption via agonists and antagonists, and population assessment (Teeter, 1980; Li et al., 2003, 2007; Sorensen and Vrieze, 2003; Twohey et al., 2003b; Buchinger et al., 2015; Sorensen, 2015). Adult trapping, however, is the only method that has been explored on a management scale (Fig. 11). Baiting existing sea lamprey traps with the male sex pheromone component 3kPZS resulted in a 10% increase in trapping efficiency on average from the status quo (Johnson et al., 2013). This modest increase in trapping efficiency has left doubts as to whether or not it is justifiable to apply 3kPZS trapping as a control tactic, but further research has shown that trapping efficiency can be further increased when the whole male pheromone (i.e. water in which sexually mature males were held that contains all pheromone components) is used as bait (Johnson et al., 2015a) and in streams with certain characteristics (e.g. wider streams with lower density adult sea lamprey populations; Johnson et al., 2015b). These positive results led to the registration of 3kPZS as a vertebrate pheromone biopesticide with regulatory agencies in the USA and Canada, the first of its kind for both countries. Management strategy evaluation modelling is now being used to help guide decisions on whether or not to add 3kPZS trapping to the sea lamprey control programme versus investing in other sea lamprey control tactics (Dawson et al., 2016). Despite the considerable knowledge gained in understanding sea lamprey chemosensory communication, a sea lamprey control tactic exploiting chemosensory cues remains elusive and further research is needed.

### Understanding genetics to manipulate critical genes

Genetic technologies have been hypothesized to hold considerable promise for the control of invasive fishes (Thresher, 2008) and were recently reviewed for sea lamprey (McCauley et al., 2015). Both the mitochondrial and nuclear genome of the sea lamprey have been sequenced (Lee and Kocher, 1995; Smith et al., 2013). With this information, researchers, especially through the use of transcriptome analysis, are now in a position to rapidly identify genes associated with key physiological functions that could be exploited for sea lamprey control purposes (McCauley et al., 2015).

One possible mechanism in which sea lamprey control could be achieved through genetic manipulation is gene knockdown. Morpholinos are one potential gene knockdown technique (McCauley et al., 2015). Morpholinos are synthetic oligonucleotides that function to block specific base-pairing surfaces of RNA. Morpholinos have been successfully used to knockdown the SoxE gene in sea lamprey, which is important for neural crest development (McCauley and Bronner-Fraser, 2006). Morpholinos could potentially be engineered to target the expression of other genes coding for specific proteins critical for sea lamprey survival. RNA interference, an endogenous process in which RNA molecules can inhibit gene expression by linking to specific mRNA, could also be exploited for sea lamprey control through gene knockdown. RNA could be synthesized to manipulate specific aspects of sea lamprey development (e.g. targeting fertility genes could produce sterile males). In fact, RNA interference as a genetic modifying technique has been demonstrated to work effectively in sea lamprey, was used to increase larval sea lamprey mortality in laboratory conditions, and was delivered via food (Heath et al., 2014).

Another gene manipulation approach that has recently advanced is exploitation of the CRISPR/Cas system (Marraffini and Sontheimer, 2010). The CRISPR/Cas system evolved as a defense mechanism in bacteria and is comprised of short, repetitive DNA base sequences that allow RNA-guided cleavage of specific DNA regions damaged by invading viruses and plasmids (Marraffini and Sontheimer, 2010; McCauley et al., 2015). The CRISPR/Cas system has been adapted to target specific genes for deletion (Cong et al., 2013) and has been applied in two lamprey species (Square et al., 2015; Zu et al., 2016).

Genetic manipulation of sex determination in sea lamprey could also prove fruitful in the search for sea lamprey control strategies. CRISPR/Cas technology has recently been developed as a gene drive (increasing the prevalence of a specific gene in a population through inheritance) to distort sex-ratios for control purposes in mosquitos (*Anopheles gambiae*; Galizi et al., 2016) This and other genetic control tactics targeting sex determination could potentially be adapted for invasive fishes (Thresher et al., 2014). As an example, the genes targeting the conversion of testosterone to estrogen are being manipulated in the common carp (*Cyprinus carpio*) to create all male offspring in hopes of eradicating local invasive populations (Thresher, 2008; McCauley et al., 2015). This ‘daughterless’ technology could potentially be adapted for sea lamprey control purposes in the future (McCauley et al., 2015).

Substantial physiological information is required before gene knockdown and ‘daughterless’ technologies can be investigated as sea lamprey control techniques, i.e. morpholinos and CRISPR require injection into embryos (McCauley et al., 2015), an unfeasible delivery mechanism for sea lamprey control. Also, ‘daughterless’ technology possesses risk of the genetic manipulation jumping to non-target populations of sea lamprey (e.g. in its native range), which could have dire consequences for the species as a whole. Risks associated with the application of genetic technologies for sea lamprey control must be vetted through ethical debate and ultimately be biologically and socially acceptable before implementation. Nevertheless, genetic technologies hold substantial promise for the development of effective, environmentally benign and species-specific sea lamprey control.

## Conclusions

The invasion of the sea lamprey into the Laurentian Great Lakes was an ecologic and economic tragedy affecting two nations and one of the world's largest fresh water ecosystems. Fortunately, the Canadian and United States governments created and continually support a successful sea lamprey control programme, which allowed rehabilitation of the Great Lakes ecosystem and economy. Valuable lessons have been learned since the inception of sea lamprey control. An intimate understanding of the sea lamprey's biology, ecology and physiology was essential for the development and refinement of effective and selective sea lamprey control tactics. Current sea lamprey control tactics—lampricides, barriers, trapping and sterile male releases—all exploit unique aspects of the sea lamprey's physiology to reduce their populations in each Great Lake. Ongoing physiological research is used to modify existing tactics and protocols to make sea lamprey control more effective, efficient and selective toward sea lamprey. Although current control tactics target several different physiological mechanisms, ongoing and future research is and will be instrumental in the pursuit of novel and innovative techniques, such as those exploiting chemosensory cues and genetics. These new techniques have the potential to further diversify the sea lamprey control programme and to be effective, efficient and more selective than current techniques. Importantly, having a diverse suite of control tactics that exploit many aspects of the sea lamprey's physiology will provide security in the event a tactic fails (e.g. if lampricide resistance evolves). Finally, physiological knowledge gained through the sea lamprey control programme could also be conversely used to inform conservation of sea lamprey in their native range where they are imperiled. Clearly, sea lamprey control in the Laurentian Great Lakes of North America is a successful example of conservation physiology.
